# 373. Closing Care Gaps: Optimizing Pre-Transplant *Trypanosoma cruzi* Testing

**DOI:** 10.1093/ofid/ofae631.114

**Published:** 2025-01-29

**Authors:** Sabrina E Newstead, Neeraja Swaminathan, Hannah Imlay

**Affiliations:** University of Utah, Salt Lake City, UT; University of Utah, Salt Lake City, UT; University of Utah Health, Salt Lake City, UT

## Abstract

**Background:**

Chronic *Trypanosoma cruzi* infection can be asymptomatic, but *T.cruzi* can reactivate and cause disease among transplant recipients following immunosuppression. Prior to transplant it is recommended to perform 2-step testing among candidates at high risk of exposure, defined as patients who were born or had prolonged residence in Mexico, Central, or South America. We reviewed transplant recipients at our institution to identify the frequency of testing, percentage with positive testing, and whether testing was being performed in the appropriate high-risk population.

**Figure 1:**
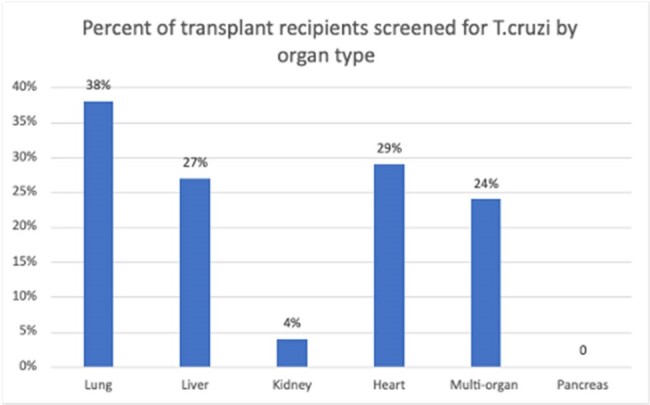
Percentage of transplant patients tested by organ type.

**Methods:**

We identified patients who underwent organ transplant from January 1, 2018 – April 18 2024 and determined how many patients underwent *T.cruzi* testing. Among those with positive tests, we identified the proportion with epidemiologic risk factors; we also determined whether appropriate confirmatory testing was obtained. We identified 40 randomly selected patients registered as Spanish-speaking in our electronic medical record (EMR), reviewed their charts to determine whether they were at high risk of exposure, and identified if they received screening.

**Figure 2:**
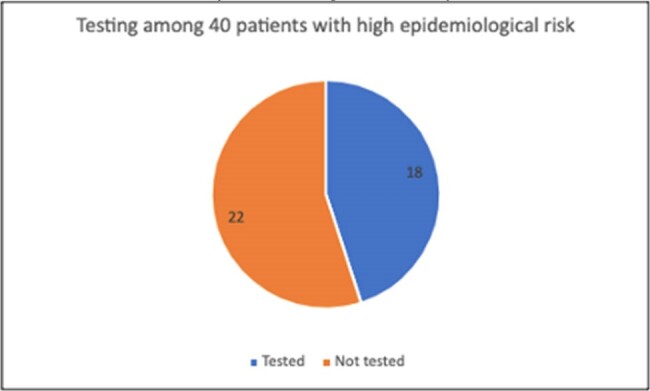
Number of patients with high epidemiologic risk who were tested vs not tested for T.cruzi, among 40 patients reviewed.

**Results:**

Among 1399 patients who underwent transplant (104 lung, 101 heart, 212 liver, 918 kidney, 62 multiorgan, 2 pancreas), 180 (12.9%) were tested for *T.cruzi* (Figure 1). Twelve of 180 tests (6.7%) were initially positive; only 8/12 (75%) positive tests were among patients with high risk for *T.cruzi*. Ten of 12 positive tests were sent for confirmatory testing and 2 tests were not sent or followed up. Only one patient had a positive confirmatory test and underwent post-transplant monitoring. Of 40 patients identified as Spanish-speaking in the EMR, all 40 had high epidemiologic risk but only 18 (45%) underwent appropriate pre-transplant *T.cruzi* testing (Figure 2).

**Conclusion:**

We identified several care gaps in our pre-transplant *T.cruzi* testing, including over-testing among patients without epidemiologic risk, under-testing among patients with risk for prior *T.cruzi* exposure, and incomplete confirmatory testing. Our data highlight an opportunity for specific testing guidance and education to optimize patient care.

**Disclosures:**

**All Authors**: No reported disclosures

